# An evaluation of the impact of missing deaths on overall survival analyses of advanced non–small cell lung cancer patients conducted in an electronic health records database

**DOI:** 10.1002/pds.4758

**Published:** 2019-03-14

**Authors:** Gillis Carrigan, Samuel Whipple, Michael D. Taylor, Aracelis Z. Torres, Anala Gossai, Brandon Arnieri, Melisa Tucker, Philip P. Hofmeister, Peter Lambert, Sandra D. Griffith, William B. Capra

**Affiliations:** ^1^ Genentech, Inc South San Francisco CA USA; ^2^ Flatiron Health New York NY USA

**Keywords:** lung cancer, missing deaths, overall survival, pharmacoepidemiology, survival analyses

## Abstract

**Purpose:**

The aim of this study was to assess the impact of missing death data on survival analyses conducted in an oncology EHR‐derived database.

**Methods:**

The study was conducted using the Flatiron Health oncology database and the National Death Index (NDI) as a gold standard. Three analytic frameworks were evaluated in advanced non‐small cell lung cancer (aNSCLC) patients: median overall survival [mOS]), relative risk estimates conducted within the EHR‐derived database, and “external control arm” analyses comparing an experimental group augmented with mortality data from the gold standard to a control group from the EHR‐derived database only. The hazard ratios (HRs) obtained within the EHR‐derived database (91% sensitivity) and the external control arm analyses, were compared with results when both groups were augmented with mortality data from the gold standard. The above analyses were repeated using simulated lower mortality sensitivities to understand the impact of more extreme levels of missing deaths.

**Results:**

Bias in mOS ranged from modest (0.6–0.9 mos.) in the EHR‐derived cohort with (91% sensitivity) to substantial when lower sensitivities were generated through simulation (3.3–9.7 mos.). Overall, small differences were observed in the HRs for the EHR‐derived cohort across comparative analyses when compared with HRs obtained using the gold standard data source. When only one treatment arm was subject to estimation bias, the bias was slightly more pronounced, but increased substantially when lower sensitivities were simulated.

**Conclusions:**

The impact on survival analysis is minimal with high mortality sensitivity with only modest impact associated within external control arm applications.

**KEY POINTS**

- The impact of missing death data on survival analyses and estimates of overall survival is small when mortality capture sensitivity is high (eg, approximately 90% or more).
- The magnitude of bias is increased and, at times, substantial, with lower mortality sensitivities in the 60% to 70% range.
- The direction of the effect estimation may also change with lower mortality sensitivities in the 60% to 70% range.
- Electronic health records mortality data with high sensitivity limit the potential for missing deaths to bias OS estimates allowing valid inferences to be drawn.

## 1 INTRODUCTION

Real‐world evidence (RWE) generated from real‐world data (RWD), including data derived from electronic health records (EHRs), is increasingly important for pharmacoepidemiological research.^1^ These data provide opportunities for deriving clinical insights and serve to complement findings from clinical trials. In order to synthesize RWD into high‐quality RWE, outcomes data are needed, and mortality‐based outcomes (eg, overall survival [OS] and progression‐free survival [PFS]) are particularly important for many disease areas, including oncology.

Mortality‐based RWD have multiple applications, both for standalone research studies and to complement traditional trials. These include describing the survival outcomes of a single group of patients (eg, median OS) and comparative effectiveness research (CER), where outcomes from two or more groups of patients are compared against each other, typically expressed as hazard ratios (HRs). Although these analyses could involve multiple data sources, a strength of RWE is that datasets are often large enough that these questions can be addressed within a single, harmonized database, which leverages consistent data‐generating mechanisms across groups. Another emerging application for RWE is to serve as an external control arm for single‐arm trials, where every patient receives the experimental treatment.^1^ Although a control arm is not built into the study, researchers often wish to make comparisons between the experimental treatment and contemporaneous control treatments external to the trial. Therefore, this application faces the additional challenge that outcomes data are compared across multiple data sources, conflating any differences in treatment effect (eg, HR estimates) with differences in underlying data. Lastly, real‐world mortality data can be used for trial planning purposes, where estimates from real‐world populations could be used for either power calculations or planning the time needed to accrue a number of events.

Due to its critical role in identifying a survival benefit associated with a treatment regimen, the quality of the underlying mortality data used in RWE studies is of salient interest. In EHR‐derived databases, mortality data are collected in a structured format as part of routine clinical care. However, researchers often must augment incomplete EHR mortality data with other sources, such as national death indices and commercial sources.^2^ Ideally, the quality of the data source will have benchmarks against a gold standard. While rules can be applied to address specificity and date agreement by flagging potential false positives or improbable dates, there are limited ways to address imperfect sensitivity short of obtaining additional data. Thus, missing deaths are of paramount interest when describing the quality of EHR‐derived mortality data.

Once mortality quality benchmarks are in place, researchers are often faced with the question of how good is good enough? There are currently no universally accepted minimum standards or consensus with regard to an acceptable level of mortality completeness; rather, the right standard likely depends on the particular analysis.^3^, ^4^ Even if deaths are missing completely at random, incomplete mortality data results in absolute estimates of OS, such as median OS (mOS), being biased upward.^5^, ^6^ If missing deaths are equally distributed between comparator arms, the impact on relative risk estimates, such as HRs, should be minimal; however, this assumption may not hold in applications such as external control arms where the experimental and control arms are drawn from different data sources. Beyond these hypothetical implications, how do missing deaths impact findings in applications of interest to researchers utilizing RWE?

We sought to answer these questions using an oncology EHR‐derived data source, where a recent study reported greater than 90% mortality sensitivity in advanced non–small cell lung cancer (aNSCLC) patients.^2^ We tested the impact of missing deaths in the EHR‐derived data source—both at the high sensitivity levels observed in practice and artificially reduced for illustrative purposes—by comparing the output of descriptive analyses, CER, and external control arms to those obtained using a gold standard data source. Thus, we aimed not only to understand the impact of missing deaths in the EHR‐derived data source utilized here but also, more broadly, to understand what levels of mortality sensitivity are high enough quality to minimize impact on analytic results.

## 2 METHODS

### 2.1 Study overview

The purpose of this retrospective observational study was to evaluate the impact of missing deaths on survival analyses from an EHR‐derived RWE database in patients diagnosed with aNSCLC, as compared with a gold standard data source. Institutional Review Board and National Center for Health Statistics approval of the study protocol was obtained prior to study conduct. Informed consent was waived as this was a non‐interventional study using routinely collected data. Flatiron Health standard methodology for data security and patient privacy was implemented.

### 2.2 Description of EHR‐derived database and gold standard data source

The Flatiron Health database contains real‐world clinical data and outcomes collected through EHRs used by cancer care providers primarily in community oncology clinics across the United States. For patients treated in the Flatiron network, information includes data entered into structured fields and contained in unstructured documents. The EHR data are subsequently linked with external mortality data. At the time of this study, the database included information from 250 cancer clinics, which consisted of approximately 775 unique sites of care in the United States, although academic centers were excluded from this analysis. The quality of mortality in the EHR‐derived database has been previously evaluated.^2^ The 2015 NDI data served as the gold standard data source. The NDI is a centralized database containing death record information from state vital statistics offices. As a result, the NDI will capture more complete death information relative to the EHR data linked with external death information for patients that have transitioned from the study's EHR network or were lost to follow up.^7^ The NDI is updated annually with death records from state vital statistics offices, so its recency does not suffice for every use case, but its completeness makes it a good historical resource for benchmarking.

### 2.3 Cohort selection and classification

Patients from the EHR‐derived database with an aNSCLC diagnosis (stage IIIB or metastatic stage IV, or recurrent advanced disease) between January 1, 2011, and December 31, 2015, were selected for inclusion to align with the data cutoff of December 31, 2015, for the gold standard data source. Patients with aNSCLC had an ICD code for lung cancer (ICD‐9 162.x or ICD‐10 C34x or C39.9), and advanced disease was confirmed via unstructured documents using “technology‐enabled abstraction,” which combines clinically trained human abstractors with software that displays portions of the chart.^8^

**Figure 1:**
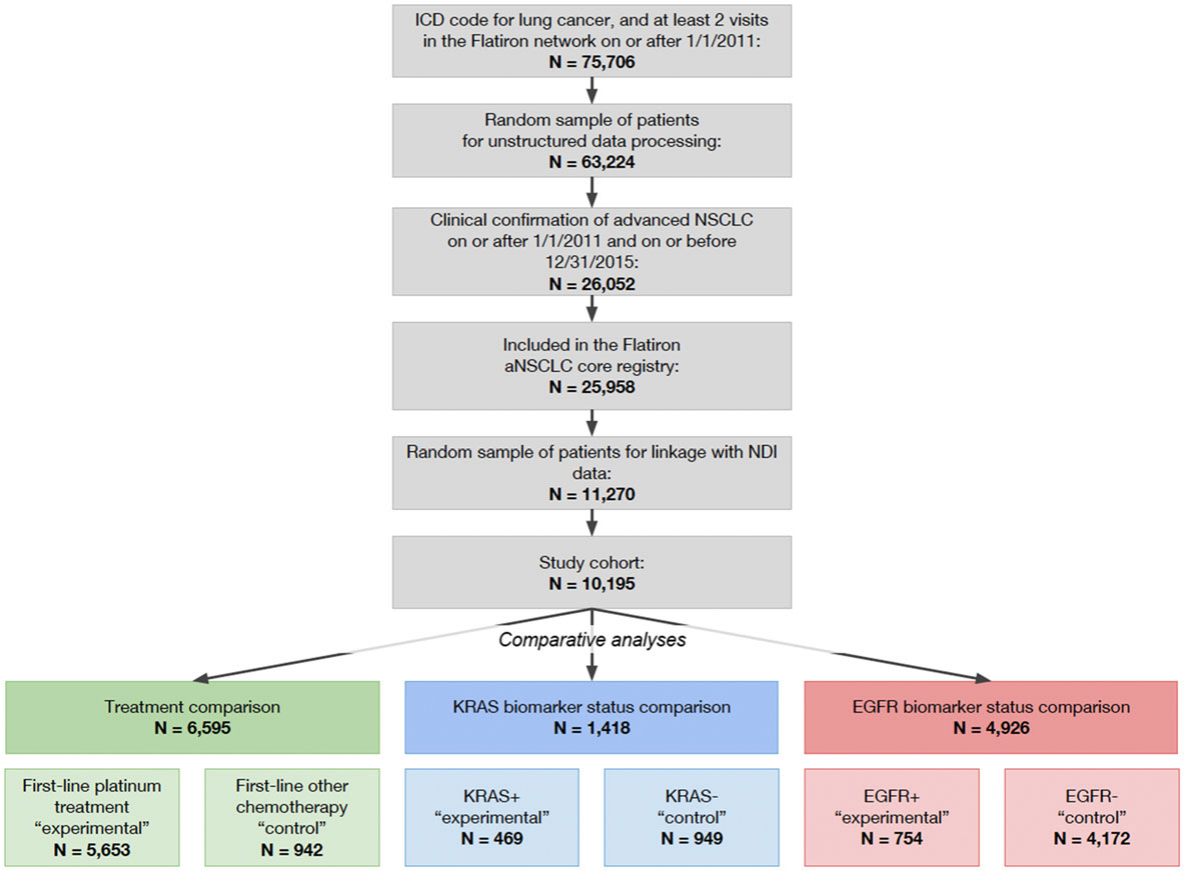
Overview of methods and analysis [Colour figure can be viewed at wileyonlinelibrary.com]

Upon linking the data sources, patients within the EHR‐derived database were classified as follows: true positives (cell A) where a death date was in both the EHR‐derived and gold standard data; false positives (cell B) where a death date was in the EHR‐derived data but not in the gold standard; false negatives (cell C) where a death date was not in the EHR‐derived data but in the gold standard; or true negatives (cell D) where no death date was in either the EHR‐derived data or in the gold standard (Figure 1, “Classification by Gold Standard”).

### 2.4 Sampling methods

In addition to the classification described above corresponding to the empirically observed sensitivity in the EHR‐derived data source (90.6% as compared with the gold standard^2^), lower sensitivity datasets were generated through simulation (Figure 1, “Sampling Methods”). Bootstrap sampling (ie, sampling with replacement) was performed with 1000 iterations to simulate sensitivities of 63.4% (simulation 1) and 72.5% (simulation 2) by randomly selecting 30% and 20% of true positives (cell A), respectively. These patients were then reclassified as false negatives (cell C) as they were no longer considered to have a death in the EHR‐derived data source. The reclassified patient groups were denoted based on the proportion of patients that were sampled from cell A and moved to cell C (ie, cells A_30_ and C_30_ for simulation 1; cells A_20_ and C_20_ for simulation 2).

### 2.5 Statistical analysis

Descriptive statistics on the demographic and clinical characteristics of the cohort were calculated, stratified by the classification of the EHR‐derived mortality variable against the gold standard. Among patients with missing EHR‐derived death data (cell C), the last confirmed structured activity date (ie, their last visit or administration in the EHR) was compared with the death date in the gold standard data source, and the distribution of differences between dates was visually examined.

Three sets of comparison groups were selected to examine the impact of missing deaths and were chosen based on known prognostic and/or predictive properties to allow for a number of expected effect sizes.^9^, ^10^, ^11^, ^12^, ^13^, ^14^ For the first comparison, we selected treatments commonly administered during the study period (2011‐2015) in the first‐line setting (ie, before the widespread use of immunotherapy) and expected to show a difference in survival between comparison groups. Patients that received a platinum‐based treatment (defined as all regimens containing cisplatin or carboplatin; “experimental group”) were compared with patients receiving other chemotherapy (defined as all regimens without a platinum agent but containing any combination of paclitaxel, nab‐paclitaxel, docetaxel, gemcitabine, vinorelbine, irinotecan, pemetrexed, or bevacizumab; “control group”). Regimens containing clinical study drugs were excluded (eg, anonymized therapies from clinical trials). The two remaining comparisons evaluated biomarker status using the patient's most recent valid test result (ie, positive or negative), as identified from unstructured documents available in the EHR, where the result was not “Results pending,” unsuccessful, or indeterminate test. One biomarker comparison was chosen based on widespread testing (EGFR+ “experimental group” versus EGFR− “control group”), and another was chosen based on less frequent testing and where there was an expected large difference in survival in order to test if the results held for a small sample size (KRAS+ “experimental group” versus KRAS− “control group”).

Using these comparison groups, three analytic use cases were evaluated: descriptive statistics of absolute risk, CER, and external control arms. For each analytic use case, a benchmark and EHR‐derived database only analysis was performed and results compared. The cohorts remained the same for each analysis, but date of death was defined according to Figure 1 (“Statistical Analysis”). Both the death date source (EHR‐derived data or gold standard data) and the subgroup of patients with a date of death (cells A + B or cells A + C) varied by analysis. The treatment and biomarker status comparisons were indexed to the initiation of first‐line treatment and advanced disease diagnosis date, respectively. If death was not observed, patients were censored at their last structured activity date.

For descriptive analyses, the primary outcome was mOS, estimated using the Kaplan‐Meier method. In CER analyses, Cox proportional hazards regression models were used to estimate HRs for death for the experimental groups, as compared with the control groups. In both descriptive and CER analytic use cases, the benchmark analyses used dates of death from the gold standard data source, and the EHR‐derived database only analyses used dates of death from the EHR‐derived data source.

Similar to the CER applications, the external control arm analytic use case also employed Cox proportional hazards regression models, but the date sources and subgroups with dates of death varied (Figure 1, “Statistical Analysis”). The benchmark analyses remained the same as the CER application—dates of death from the gold standard data for both comparison groups—intended to replicate a two‐arm clinical trial with near‐perfect mortality outcome data. The EHR‐derived database only analyses, however, utilized dates of death from different sources for each comparison group. The experimental arm utilized dates of death from the gold standard data source for patients in cells A + C, aiming to replicate a single‐arm clinical trial with near‐perfect mortality outcome data. The control arm utilized dates of death from the EHR‐derived data source for patients in cells A + B, aiming to replicate an external control arm sourced from EHR‐derived data. HRs were estimated for each pair of comparison groups.

All analytic use cases were replicated with the simulated, lower sensitivity datasets, substituting cells A and C with cells A_30_ and C_30_ (simulation 1) and cells A_20_ and C_20_ (simulation 2), as described in the sampling methods section. If death was not observed in the simulated dataset, reclassified patients were censored at a randomly assigned censor date between their advanced diagnosis date and death date from the gold standard data source. All other patients were censored at the last structured activity date in the EHR. For each simulated sensitivity, mOS and HRs were calculated for each of the 1000 iterations and the median was selected as the point estimate, with the 97.5 and 2.5 percentiles as the upper and lower confidence interval (CI) bounds.

## 3 RESULTS

**Figure 2:**
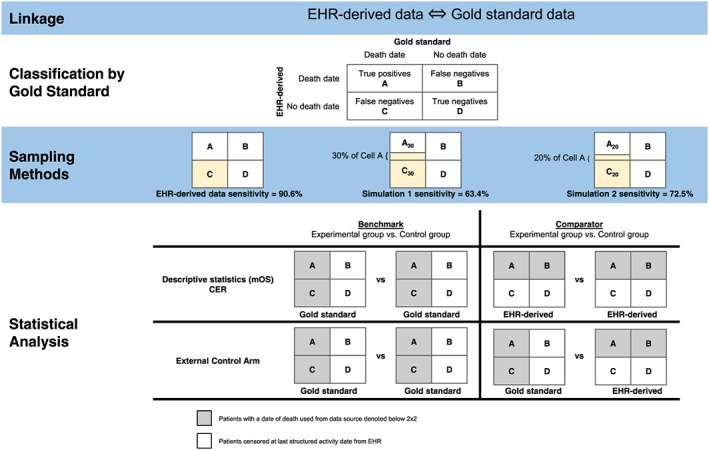
Attrition diagram [Colour figure can be viewed at wileyonlinelibrary.com]

A total of 10 195 aNSCLC patients were available for this analysis (Figure 2). Table 1 provides descriptive statistics, stratified by classification according to the gold standard data source. Comparisons among the different categories revealed modest differences between groups. When comparing patients with missing death dates (ie, false negatives, cell C) to other patients, the group with missing death dates had a lower proportion of white patients and a higher proportion of black patients; more patients residing in the western region with less in both the northeast and midwestern regions; a higher proportion of patients with no treatment recorded in the EHR; and a shorter median follow‐up time recorded in the EHR. Differences in the race distribution could reflect reporting variations across race while it would be expected that patients with missing death dates would also be associated with both less treatment (eg, reflecting patients with incomplete EHR records or patients treated outside of the EHR) and shorter follow‐up time due to mortality.

**Table 1:** Demographic and clinical characteristics of aNSCLC patients

Clinical or Demographic Characteristic	A N = 6157	B N = 136	C N = 639	D N = 3263
Age categories (binary) at advanced diagnosis				
<65 y	1931 (31.4%)	32 (23.5%)	196 (30.7%)	1164 (35.7%)
65+ y	4226 (68.6%)	104 (76.5%)	443 (69.3%)	2099 (64.3%)
Gender				
Female	2706 (43.9%)	69 (50.7%)	302 (47.3%)	1727 (52.9%)
Male	3451 (56.1%)	67 (49.3%)	336 (52.6%)	1536 (47.1%)
Unknown	0 (0.00%)	0 (0.00%)	1 (0.16%)	0 (0.00%)
Race/Ethnicity				
White	3998 (78.9%)	80 (67.2%)	340 (65.8%)	2133 (75.7%)
Black or African American	414 (8.17%)	8 (6.72%)	71 (13.7%)	258 (9.16%)
Asian	90 (1.78%)	10 (8.40%)	19 (3.68%)	116 (4.12%)
Other race	567 (11.2%)	21 (17.6%)	87 (16.8%)	310 (11.0%)
Region				
Northeast	1679 (27.3%)	33 (25.0%)	112 (18.3%)	944 (29.4%)
Midwest	1198 (19.5%)	33 (25.0%)	66 (10.8%)	589 (18.3%)
South	2394 (39.0%)	37 (28.0%)	243 (39.6%)	1143 (35.6%)
West	868 (14.1%)	29 (22.0%)	192 (31.3%)	536 (16.7%)
Validation period (y)				
2011	333 (5.41%)	7 (5.15%)	17 (2.66%)	9 (0.28%)
2012	920 (14.9%)	15 (11.0%)	64 (10.0%)	34 (1.04%)
2013	1401 (22.8%)	34 (25.0%)	137 (21.4%)	76 (2.33%)
2014	1672 (27.2%)	42 (30.9%)	211 (33.0%)	150 (4.60%)
2015	1831 (29.7%)	38 (27.9%)	210 (32.9%)	2994 (91.8%)
Histology				
Non–squamous cell carcinoma	4124 (67.0%)	98 (72.1%)	445 (69.6%)	2339 (71.7%)
Squamous cell carcinoma	1586 (25.8%)	31 (22.8%)	147 (23.0%)	779 (23.9%)
NSCLC histology NOS	447 (7.26%)	7 (5.15%)	47 (7.36%)	145 (4.44%)
Group stage at diagnosis				
Stage I/II	685 (11.1%)	12 (8.82%)	65 (10.2%)	563 (17.3%)
Stage III/IIIA	539 (8.75%)	7 (5.15%)	62 (9.70%)	289 (8.86%)
Stage IIIB/IV	4608 (74.8%)	112 (82.4%)	478 (74.8%)	2261 (69.3%)
Group stage is not reported	325 (5.28%)	5 (3.68%)	34 (5.32%)	150 (4.60%)
Smoking status				
History of smoking	5307 (86.2%)	113 (83.1%)	521 (81.5%)	2704 (82.9%)
No history of smoking	608 (9.87%)	22 (16.2%)	92 (14.4%)	509 (15.6%)
Unknown/not documented	242 (3.93%)	1 (0.74%)	26 (4.07%)	50 (1.53%)
ALK status^a^				
Rearrangement not present	2390 (89.3%)	62 (91.2%)	220 (86.6%)	1535 (89.1%)
Rearrangement present	54 (2.02%)	1 (1.47%)	7 (2.76%)	73 (4.24%)
Unsuccessful/indeterminate test	194 (7.25%)	4 (5.88%)	23 (9.06%)	97 (5.63%)
Unknown	37 (1.38%)	1 (1.47%)	4 (1.57%)	17 (0.99%)
EGFR status^a^				
Mutation negative	2489 (84.1%)	53 (70.7%)	235 (80.5%)	1395 (73.9%)
Mutation positive	294 (9.93%)	17 (22.7%)	42 (14.4%)	401 (21.2%)
Unsuccessful/indeterminate test	150 (5.07%)	4 (5.33%)	13 (4.45%)	83 (4.40%)
Unknown	28 (0.95%)	1 (1.33%)	2 (0.68%)	9 (0.48%)
ROS1 status^a^				
Rearrangement not present	480 (88.7%)	10 (90.9%)	46 (83.6%)	633 (90.8%)
Rearrangement present	12 (2.22%)	1 (9.09%)	1 (1.82%)	7 (1.00%)
Unsuccessful/indeterminate test	46 (8.50%)	0 (0.00%)	6 (10.9%)	50 (7.17%)
Unknown	3 (0.55%)	0 (0.00%)	2 (3.64%)	7 (1.00%)
KRAS status^a^				
Mutation negative	504 (64.2%)	10 (55.6%)	57 (63.3%)	378 (64.2%)
Mutation positive	248 (31.6%)	5 (27.8%)	28 (31.1%)	188 (31.9%)
Unsuccessful/indeterminate test	33 (4.20%)	3 (16.7%)	5 (5.56%)	23 (3.90%)
PDL1 status^a^^,^ b				
PD‐L1 negative/not detected	23 (52.3%)	0 (0.00%)	3 (60.0%)	73 (54.5%)
PD‐L1 positive	21 (47.7%)	2 (100%)	2 (40.0%)	50 (37.3%)
Unsuccessful/indeterminate test	0 (0.00%)	0 (0.00%)	0 (0.00%)	8 (5.97%)
No interpretation given in report	0 (0.00%)	0 (0.00%)	0 (0.00%)	3 (2.24%)
Gap between systemic therapy and advanced diagnosis				
No treatment	1527 (24.8%)	30 (22.1%)	220 (34.4%)	846 (25.9%)
No, ≤90‐d gap	3854 (62.6%)	86 (63.2%)	343 (53.7%)	1924 (59.0%)
Yes, >90‐d gap	776 (12.6%)	20 (14.7%)	76 (11.9%)	493 (15.1%)
Gap between structured activity and advanced diagnosis				
No activity after advanced diagnosis date	50 (0.81%)	0 (0.00%)	7 (1.10%)	37 (1.13%)
No, ≤90‐d gap	5443 (88.4%)	121 (89.0%)	552 (86.4%)	2649 (81.2%)
Yes, >90‐d gap	664 (10.8%)	15 (11.0%)	80 (12.5%)	577 (17.7%)
Received platinum‐based 1L therapy: Yes	3497 (56.8%)	78 (57.4%)	321 (50.2%)	1757 (53.8%)
Received EGFR‐targeted 1L therapy: Yes	396 (6.43%)	14 (10.3%)	37 (5.79%)	301 (9.22%)
Received other chemotherapy in 1L: Yes	647 (10.5%)	11 (8.09%)	51 (7.98%)	233 (7.14%)
Follow‐up time from advanced diagnosis (mo), median [IQR]	5.36 [2.04‐11.6]	5.64 [2.53‐13.6]	4.37 [1.55‐10.9]	10.3 [3.95‐22.2]

a Among those tested and based upon most recent successful biomarker test.

b PD‐L1 “Unsuccessful/indeterminate test” results also include “PD‐L1 equivocal” results.

**Figure 3:**
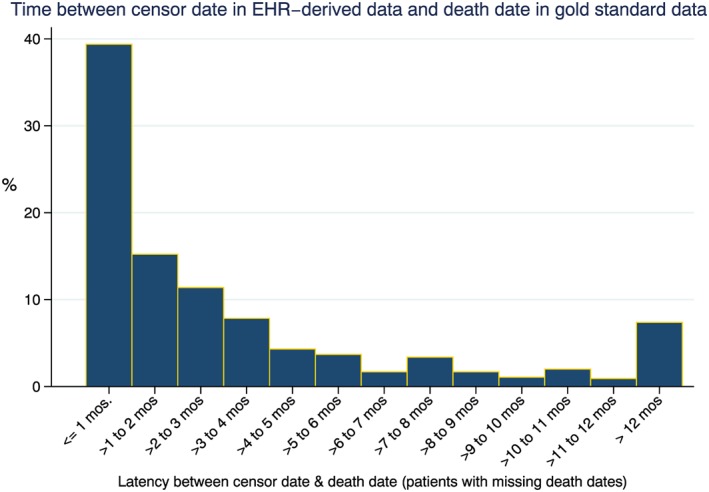
Censoring patterns of those patients with missing death dates (cell C false‐negative patients) [Colour figure can be viewed at wileyonlinelibrary.com]

Figure 3 depicts the relationship between censor date in the EHR‐derived data and death date in the gold standard data source in patients with missing death dates. A high proportion of patients with missing deaths in the EHR‐derived database had a last structured activity date that was within 1 month (39%) and 4 months (74%) of their death date in the gold standard data source. The clustering between the last activity date and the death date identified in the NDI suggests that censoring of these patients is nonrandom and instead signifies unidentified death events in the EHR.

### 3.1 Impact of missing deaths on absolute survival estimates

An upward bias in mOS of approximately 0.5 months was observed in EHR‐derived death data for patients treated with platinum agents in first‐line compared with the benchmark (Table 2). For the simulated cohorts with mortality sensitivities of 63.4% and 72.5%, the bias increased substantially to 3.3 and 2.2 months, respectively. A similar trend was observed in patients treated with other chemotherapy, as well as the biomarker‐based groups. Overall, the magnitude of bias in mOS comparing the gold standard data source with the EHR‐derived death data ranged from approximately 2.5% to 8.1%, while the bias observed in the simulated lower mortality sensitivities ranged from 36.7% to 53.2% for simulation 1. Observed differences in mOS for KRAS+ were as much as 9.7 months in simulation 1 (63.4% mortality sensitivity) and 6.2 months in simulation 2 (72.5% mortality sensitivity).

**Table 2:** Impact of missing deaths on measures of absolute risk (mOS)

Median Overall Survival and 95% CI, mo							
Exposure Group	Simulation 1 (63.4%)	Bias, %	Simulation 2 (72.5%)	Bias, %	EHR‐derived (90.6%)	Bias, %	Gold Standard Data
Platinum treatment	12.3 (11.7‐13.0)	36.7	11.2 (10.7‐11.7)	24.4	9.5 (9.1‐9.9)	5.6	9.0 (8.7‐9.4)
Other Chemo	11.8 (10.1‐13.6)	53.2	10.1 (8.8‐11.9)	31.2	8.2 (7.3‐9.6)	6.5	7.7 (6.9‐8.7)
EGFR+	20.7 (17.8‐25.9)	46.8	19.4 (15.9‐22.2)	37.6	15.0 (13.4‐18.9)	6.4	14.1 (12.1‐17.3)
EGFR‐	21.2 (18.9‐25.3)	43.2	19.2 (17.1‐21.4)	29.7	16.0 (14.5‐18.3)	8.1	14.8 (14.0‐17.0)
KRAS+	33.4 (28.5‐NA)	40.9	29.9 (24.9‐36.2)	25.8	24.3 (22.3‐29.4)	2.5	23.7 (21.7‐27.3)
KRAS‐	16.3 (15.2‐17.2)	39.3	14.6 (13.9‐15.3)	24.8	12.4 (11.8‐13.1)	6.0	11.7 (11.3‐12.4)

### 3.2 Impact on CER analyses

**Figure 4:**
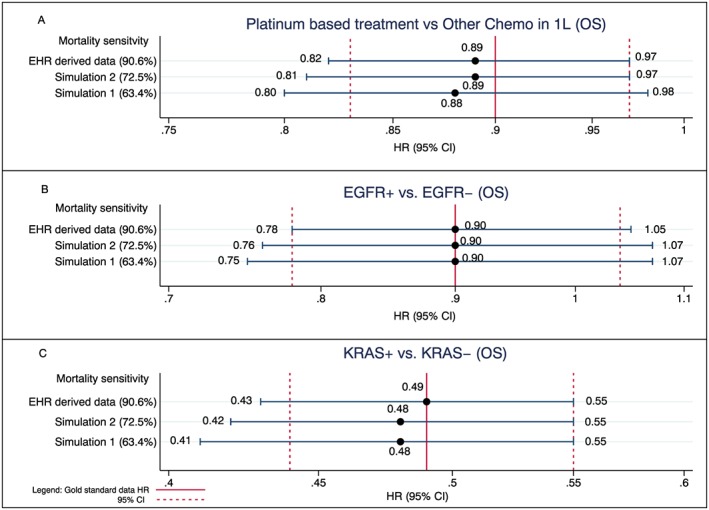
Impact of missing deaths on comparative analyses conducted with EHR‐derived data: current mortality sensitivity vs simulated sensitivities compared with gold standard benchmark [Colour figure can be viewed at wileyonlinelibrary.com]

When HRs were examined, a lack of systematic bias between exposure groups was observed, including within the two simulated cohorts, with only small differences seen in the HRs for the EHR‐derived death data across all three comparisons relative to HRs obtained for the gold standard death data (Figure 4A‐C). Despite large observed differences in mOS between the simulated cohorts and the gold standard cohort, comparisons of relative risk such as HRs were largely unaffected by missing death data.

### 3.3 Impact on external control arm analyses

**Figure 5:**
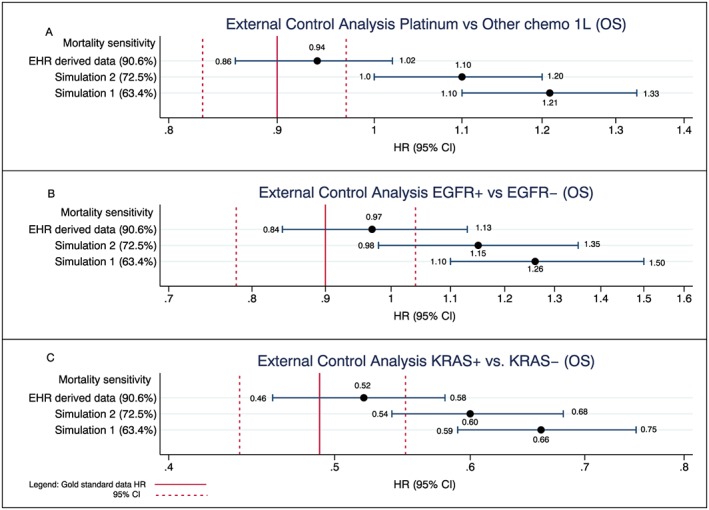
Impact of missing deaths on analyses that use the EHR‐derived data as an external control arm: current mortality sensitivity vs simulated sensitivities compared with gold standard benchmark. For the external control analyses, the experimental arm in all analyses is composed of the gold standard data, and the control arm is composed of the EHR‐derived data only. For simulations 1 and 2, the same approach is taken where the experimental arms are composed of the gold standard data, and the control arms are composed of the EHR‐derived data only (with their respective simulated lower sensitivities). Each analysis is in turn compared with an analysis conducted using the gold standard data only (solid red vertical line in Figure 5 represents the HR using the gold standard with dashed line representing its corresponding 95% CI) [Colour figure can be viewed at wileyonlinelibrary.com]

Given the systematic differences in mortality capture of the exposure groups *artificially* introduced in the external control arm analyses, the impact of missing deaths on these analyses was much more pronounced (Figure 5A‐C) than in analyses performed entirely within the EHR‐derived database (ie, measures of absolute risk or CER analyses). The impact ranged from modest differences in the EHR‐derived data to much more pronounced differences in the two simulated cohorts. For example, in the EGFR biomarker analysis, a slight bias towards the null was observed in the EHR‐derived database: HR = 0.97 (95% CI, 0.84‐1.13) compared with an HR = 0.90 for the gold standard. However, a much greater bias was observed in both simulated analyses with an HR of 1.26 (95% CI, 1.10‐1.50) observed in the simulated analysis with 63.4% sensitivity and an HR of 1.15 (95% CI, 0.98‐1.35) in the simulated 72.5% sensitivity when compared with the gold standard, which, again, had an HR of 0.90 (95% CI, 0.78‐1.04). The same effects were observed in the other use cases (eg, platinum vs other chemo and KRAS+ vs KRAS−).

## 4 DISCUSSION

RWE sources, including EHR‐derived datasets, are valuable analytic platforms for conducting clinical research.^1^ Mortality serves as the primary outcome in many analyses across disease areas and particularly for oncology. However, it is often incomplete because of imperfect data collection systems, workflows not designed to capture mortality data, and patients lost to follow up.^5^, ^15^, ^16^ The purpose of this study was to examine the potential impact of missing death data in an EHR‐derived oncology data source, which is of critical importance to establishing a research‐grade EHR‐derived database and should provide guidance with respect to an acceptable level of completeness.^16^, ^17^, ^18^

In CER analyses, there was little to no impact on the estimated HRs as compared with the gold standard data source, regardless of the sensitivity level. This result suggests that conclusions from CER analyses where both comparators originate from the same high‐sensitivity RWE data source can be interpreted with confidence, even for the lower sensitivity data sources simulated here. However, data sources with missing deaths consistently overestimated mOS, as compared with the gold standard data source. This impact was modest for the sensitivity observed in the EHR‐derived database (2.5%‐8.1% bias) but increased when sensitivity was artificially lowered (eg, up to 53.2% bias in the simulated cohort with 63.4% sensitivity). These findings have implications for descriptive analyses and trial planning. For trial planning, event accrual estimates based upon mOS would be conservative (events would accrue more quickly than predicted); however, when mOS estimates are presented descriptively, caution in their interpretation is warranted, particularly when sensitivity is low.

One key opportunity for RWE in drug development is to serve as an external control for a single‐arm clinical trial.^1^ With the EHRs current mortality sensitivity of greater than 90%, creating differential sensitivity in the context of external control arm analyses resulted only in small differences in estimated HRs and would lead to conservative conclusions and biasing against the experimental arm (eg, absolute change in HR of less than 0.05 towards the null) with corresponding small increases in the probability of type II errors. In drug development, bias in the direction of the null is preferable to an enhanced risk of a type I error. Also, decisions on molecule phase advancement within drug development (e.g., from single‐arm phases 1b to 3 randomized trials) generally would not change based on a 0.05 absolute difference in a phase 1b HR. Conversely, when the sensitivity was lowered, the impact was far more pronounced and much more likely to alter decision making.

Other studies have examined the impact of missing deaths with little evidence to suggest meaningful estimation bias when the mortality outcome is reasonably well captured (ie, 85%‐90% sensitivity).^4^, ^15^ Some studies have observed systematic differences in comparative analyses likely attributable in part to informative censoring in settings where exposures are related differentially to the mortality outcome.^16^, ^19^ Given the absence of any meaningful estimation bias when sensitivity is greater than 90%, why was it important to conduct this study? It was clear from the simulated analyses at lower sensitivities that the impact of missing endpoints such as mortality can have a major effect on analyses, in particular on estimates of absolute risk. Although there are a number of thresholds that have been discussed with respect to levels of missing outcomes in EHRs, there is a dearth of empirical support.^3^, ^4^ Understanding the impact of missing deaths in EHRs is essential to instilling confidence in this rapidly evolving source of clinical evidence. In doing so, researchers will ensure a level of scientific rigor that will allow for sensible use of EHR‐derived data for clinical research as an adjunct to the gold standard randomized clinical trials.

There are a number of study limitations that should be considered when evaluating the findings. First, this study leveraged data from community‐based oncology clinics in the United States, and patterns of missing data may be different in academic centers or in other countries. This analysis assumes that the NDI is a gold standard for mortality, yet any database at this scale is unlikely to capture every death.^20^ Second, this study did not consider the mechanism for missing deaths. Although we observed little impact on the examples studied here with high‐sensitivity mortality data, regardless of mechanism, further work is needed to describe the presence and degree of informative censoring in these data and understand its impact. Third, despite the minimal impact on most conclusions observed in aNSCLC, it is unclear how this will expand to other cancer types with longer mOS. Lastly, although the comparison groups were chosen to represent a range of common research questions, they are not exhaustive.

Strength of the study include the varying levels of mortality sensitivity and large sample size. Additionally, the study utilized a gold standard data source; in many examinations of missing data, a proxy for the complete data is never available for comparison. Finally, a variety of analytic use cases were examined, including the novel use of EHR‐derived data as an external control.

Although modest bias was observed for absolute estimates and external control analyses when sensitivity was greater than 90%, the bias occurred in a consistent direction and would not likely impact study conclusions or decision making. However, mortality data with lower sensitivity allows for the possibility of more substantial bias to enter into analyses conducted using EHR‐derived data. For analyses of mortality based on external controls, researchers should understand the level sensitivity of the data and consider the impact on bias. Using EHR‐derived mortality data with high sensitivity mitigates the likelihood that analyses performed using the data will be subject to bias of any meaningful magnitude. In fact, based on the findings from the current study, achieving perfect mortality capture (100% sensitivity) in an EHR‐derived database would not result in meaningful gains in terms of a researcher's ability to draw conclusions from the data as compared with the greater than 90% sensitivity observed in this dataset.

## ETHICS STATEMENT

Institutional Review Board approval of was obtained prior to study conduct, and included a waiver of informed consent.

## CONFLICT OF INTEREST

Anala Gossai, Aracelis Z. Torres, Sandra D. Griffith, and Melisa Tucker are employees of Flatiron Health, a member of the Roche Group. Flatiron is a cancer‐focused health technology company in New York, NY, and the source of study data. Brandon M. Arnieri, Samuel A. Whipple, William B. Caprai, Michael D. Taylor, Peter Lambert, and Gillis Carrigan are employees of F. Hoffmann‐La Roche AG. Roche is a healthcare company that operates worldwide. Philip Hofmeister declares no conflict of interest.

## References

[1] Khozin S , Blumenthal GM , Pazdur R . Real‐world data for clinical evidence generation in oncology. JNCI J Natl Cancer Inst. 2017;109(11). 10.1093/jnci/djx187 29059439

[2] Curtis MD , Griffith S , Tucker M , et al. Development and validation of a high‐quality composite real‐world mortality endpoint. Health Serv Res. 2018;4‐17. 10.1111/1475-6773.12872 PMC6232402 29756355

[3] Wu YX , Johanna J , Takkenberg M , Grunkemeier GL . Measuring follow‐up completeness. Ann Thorac Surg. 2008;85:1155‐1157. 18355488 10.1016/j.athoracsur.2007.12.012

[4] Wu YX , Furnary AP , Grunkemeier GL . Using the National Death Index to validate the noninformative censoring assumption of survival estimation. Ann Thorac Surg. 2008;85:1256‐1260. 18355506 10.1016/j.athoracsur.2007.12.013

[5] Joseph G , Ibrahim HC , Chen M‐H . Missing data in clinical studies: issues and methods. J Clin Oncol. 2012;30(26). 10.1200/JCO.2011.38.7589 PMC3948388 22649133

[6] Siannis F . Sensitivity analysis for multiple right censoring processes: investigating mortality in psoriatic arthritis. Stat Med. 2011;30:356‐367. 21225898 10.1002/sim.4117

[7] National Death Index . National Center for Health Statistics, Centers for Disease Control and Prevention, Department of Health and Human Services; 2009.

[8] Liede A , Hernandez RK , Roth M , Calkins G , Nicacio L , Larrabee K . Validation of International Classification of Diseases coding for bone metastases in electronic health records using technology‐enabled abstraction. Clin Epidemiol. 2015;7:441‐448. 10.2147/CLEP.S92209. eCollection 2015 26635485 PMC4646479

[9] Marabese M , Ganzinelli M , Garassino MC , et al. KRAS mutations affect prognosis of non‐small‐cell lung cancer patients treated with first‐line platinum containing chemotherapy. Oncotarget. 6(32). 10.18632/oncotarget.5607 PMC4741822 26416458

[10] Meert AP , Martin B , Delmotte P , et al. The role of EGFR expression on patient survival in lung cancer: a systematic review with meta‐analysis. Eur Respir J. 2002;20:975‐981. 12412692 10.1183/09031936.02.00296502

[11] Shepherd FA , Rodrigues Pereira J , Ciuleanu T , et al. Erlotinib in previously treated non‐small cell lung cancer. N Engl J Med. 2006;353:123‐132.7. 10.1056/NEJMoa050753 16014882

[12] Tsao M‐S , Sakurada A , Cutz J‐C , et al. Erlotinib in lung cancer—molecular and clinical predictors of outcome. N Engl J Med. 2006;353:133‐144. 10.1056/NEJMoa050736 16014883

[13] Souquet PJ , Chauvin F , Boissel JP , et al. Polychemotherapy in advanced non‐small cell lung cancer: a meta‐analysis. Lancet. 1993;342(8862):19‐21. 8100290 10.1016/0140-6736(93)91882-m

[14] Non‐Small Cell Lung Cancer Collaborative Group . Chemotherapy in non‐small cell lung cancer: a meta‐analysis using updated data on individual patients from 52 randomized trials. BMJ. 1995;311:899‐909. 7580546 PMC2550915

[15] Pinheiro PS , Morris CR , Liu L , Bungum TJ , Altekruse SF . The impact of follow‐up type and missed deaths on population‐based cancer survival studies for Hispanics and Asians. J Natl Cancer Inst Monogr. 2014;2014(49):210‐217. 25417234 10.1093/jncimonographs/lgu016 PMC4841164

[16] Campigotto F , Weller E . Impact of informative censoring on the Kaplan‐Meier estimate of progression‐free survival in phase II clinical trials. J Clin Oncol. 2014;2014(32):3068‐3074. 10.1200/JCO.2014.55.6340 PMC4162501 25113767

[17] Boak MB , M'ikanatha NM , Day RS , Harrison LH . Internet death notices as a novel source of mortality surveillance data. Am J Epidemiol. 2008;167:532‐539. 18079132 10.1093/aje/kwm331

[18] Perkins NJ , Cole SR , Harel O , et al. Principled approaches to missing data in epidemiologic studies. Am J Epidemiol. 2018;187(3):568‐575. 29165572 10.1093/aje/kwx348 PMC5860376

[19] Rothmann M , Koti K , Lee KY , et al. Evaluating and adjusting for premature censoring of progression‐free survival. J Biopharm Stat. 2013;23(5):1091‐1105. 23957518 10.1080/10543406.2013.813526

[20] Lash TL , Silliman RA . A comparison of the National Death Index and Social Security Administration databases to ascertain vital status. Epidemiology. 2001;12(2):259‐261. 11246590 10.1097/00001648-200103000-00021

